# Genome-Wide Identification and Biotic Stress Responses of *TLP* Gene Family in *Citrus sinensis*

**DOI:** 10.3390/ijms262010133

**Published:** 2025-10-18

**Authors:** Xingtao Li, Lizhen Fan, Chang Liu, Xinrui Wang, Xiaoyuan Zhang, Xiaonan Tong

**Affiliations:** National Navel Orange Engineering Research Center, College of Life Sciences, Gannan Normal University, Ganzhou 34100, China

**Keywords:** *Citrus sinensis*, *TLP* gene family, phylogenetic analysis, biotic stress, expression analysis

## Abstract

Thaumatin-like proteins (TLPs), a subfamily of pathogenesis-related (PR) proteins, play a vital role in plant defense against pathogens. In this study, 23 *CsTLP* genes were identified in the *Citrus sinensis* genome. These genes encode proteins ranging from 203 to 512 amino acids, with molecular weights between 21.88 and 53.75 kDa, classifying them as small molecular weight proteins. The *CsTLP* genes are unevenly distributed across eight chromosomes, with chromosome 3 containing the highest number (6 genes). Subcellular localization predictions indicate that most *CsTLPs* are located in the extracellular space. Phylogenetic analysis with *Arabidopsis thaliana TLPs* classified the *CsTLPs* into 10 clades, with clade 5 being the largest. Three segmentally duplicated gene pairs were identified, suggesting a mechanism for the expansion of this gene family. Expression profiling revealed tissue-specific patterns, with the highest expression levels observed in roots and leaves. Under biotic stress, qRT-PCR analysis of 12 selected *CsTLPs* demonstrated pathogen-specific responses: *CsTLP9* and *CsTLP22* were strongly upregulated during Huanglongbing (HLB, bacterial) infection, by 21.70-fold and 9.47-fold, respectively. Multiple genes, including *CsTLP5*/*13*/*18*/*21*/*23*, exhibited over 10-fold upregulation following Citrus Anthracnose (CA, fungal) infection; however, most genes showed only weak responses to Citrus tristeza virus (CTV, viral). These findings underscore the regulatory significance of *CsTLPs* in pathogen responses and provide an important theoretical foundation for enhancing molecular disease-resistance breeding in *Citrus sinensis*.

## 1. Introduction

In the co-evolution of plants and pathogens, plants have developed complex and efficient defense mechanisms to resist pathogenic infections [1,2]. A key component of these disease resistance mechanisms is the activation of various defense responses, including the production of Pathogenesis-Related (PR) proteins [3]. Initially identified in tobacco plants infected with tobacco mosaic virus, PR proteins are now known to be widely distributed across animals, plants, and fungi [4]. Their expression is significantly induced by infection with fungi or bacteria, or in response to certain chemical stimuli [4,5]. PR proteins are closely associated with systemic acquired resistance (SAR) and the hypersensitive response (HR), serving as major products of plant defense genes and playing crucial roles in immune defense through functions such as toxin degradation, viral capsid inhibition and direct pathogen attack [3,4,5]. Based on amino acid sequence, serological properties, enzymatic activity, and functional characteristics, PR proteins are categorized into 17 families, including glucanases, chitinases, peroxidases, defensins, and thaumatin-like proteins [5].

Thaumatin-like protein (TLP), also designated as PR5, is a member of the PR protein family and typically comprises around 200 amino acids [6,7]. It derives its name from its high sequence similarity to thaumatin, a sweet-tasting protein from the West African shrub *Thaumatococcus daniellii* [8]. A conserved structural motif in TLP is the core region: G-X-[GF]-X-C-X-T-[GA]-D-C-X-(1,2)-G-X-(2,3)-C, along with a distinctive REDDD motif composed of arginine, glutamic acid, and three aspartic acids [9,10]. TLPs also contain 10 or 16 conserved cysteine residues that form 5 or 8 disulfide bonds, which confer resistance to extreme pH, high temperatures, and proteolytic degradation [11,12]. The tertiary structure of TLP primarily consists of three domains: Domain I, Domain II, and a “V”-shaped cleft located between Domains I and II. This cleft, which may be acidic, neutral, or basic, is proposed to bind various ligands or receptors [13]. Studies have indicated that the crystal structure of acidic PR-5 subtypes exhibits a distinctive deep and acidic cleft on the surface, which is critical for their antifungal activity [4].

*TLPs* significantly contribute to plant resistance against biotic stresses, providing defense against insects, fungi, bacteria, and viruses [14]. They are also involved in responses to various abiotic stresses such as high temperature, drought, cold, salinity, and ultraviolet radiation [14]. Beyond stress adaptation, *TLPs* function in diverse biological processes including seed germination, floral development, fruit maturation, senescence, and glucanase regulation [15]. Among these roles, their response to fungal pathogens is the most thoroughly investigated. *TLP* gene expression is induced by infection with various fungi, including *Colletotrichum gloeosporioides* [16], *Verticillium dahliae* [17], *Fusarium solani* [18], *Rhizoctonia cerealis* [19]. Transgenic plants overexpressing *TLPs* exhibit delayed disease progression and broad-spectrum resistance [20]. Studies confirm that TLP isoforms from multiple plant sources display significant glucanase activity, facilitating the hydrolysis of β-D-glucan, a key structural component of the cell wall in most oomycetes [4,17]. Furthermore, TLPs can inhibit xylanase, α-amylase, and trypsin, thereby contributing to the disruption of fungal cell membranes and spores, reducing the viability of germinating spores, and inducing programmed cell death in fungi [21,22].

Citrus holds a vital position in the global fruit market and represents a major economic crop. Sweet orange (*Citrus sinensis*), in particular, is valued for its nutritional quality and popular for its juicy, sweet fruit [23]. However, its cultivation faces challenges from pathogen attacks and environmental stressors. Globally, diseases such as Citrus Anthracnose (CA), Citrus Huanglongbing (HLB) and Citrus tristeza virus (CTV) threaten citrus production [24,25,26]. These diseases impair tree growth, reduce yield, and compromise fruit quality, leading to substantial economic losses [25,27,28]. A comprehensive analysis of the *TLP* gene family in *Citrus sinensis* and its functional role in biotic stress response will provide critical insights for future genetic improvement of disease resistance. In this study, we identified *TLP* genes in the *Citrus sinensis* genome using bioinformatic tools and characterized their physicochemical properties, gene structures, protein domains, phylogenetic relationships, collinearity, and *cis*-regulatory elements. Using qRT-PCR, we also investigated the expression profiles of *TLP* genes under infection with HLB, CTV, and CA, to elucidate their functional roles and support their application in breeding disease-resistant citrus varieties.

## 2. Results

### 2.1. Identification and Physicochemical Property Analysis of CsTLP Gene Family

In the *Citrus sinensis* genome, 23 *TLP* genes were identified and systematically named *CsTLP1* to *CsTLP23* according to their chromosomal locations (Table S1). Analysis of their physicochemical properties (Table 1 and Table S2) showed that the protein lengths vary from 203 aa (CsTLP10) to 512 aa (CsTLP22), with molecular weights ranging from 21.88 kDa (CsTLP10) to 53.75 kDa (CsTLP22), and an average of 28.41 kDa. Except for *CsTLP6*/*8*/*9*/*13*/*14*/*16*/*19*/*21*, all other *CsTLPs* exhibit theoretical pI values below 7. The instability index ranges from 24.13 (CsTLP14) to 57.99 (CsTLP15), with 14 out of the 23 proteins scoring above 40, indicating that most CsTLPs are unstable. The aliphatic index varies between 55.74 (CsTLP18) and 78.48 (CsTLP13). Hydropathicity analysis showed that 8 CsTLPs (CsTLP1/5/6/7/9/16/19/23) display positive GRAVY values, while the rest are negative, suggesting the coexistence of hydrophilic and hydrophobic regions. Subcellular localization predictions indicate that *CsTLP14* is vacuolar, while the other 22 are extracellular.

### 2.2. Chromosome Distribution, Gene Structure and Conserved Motif Analysis of the CsTLP Gene Family

**Figure 1:**
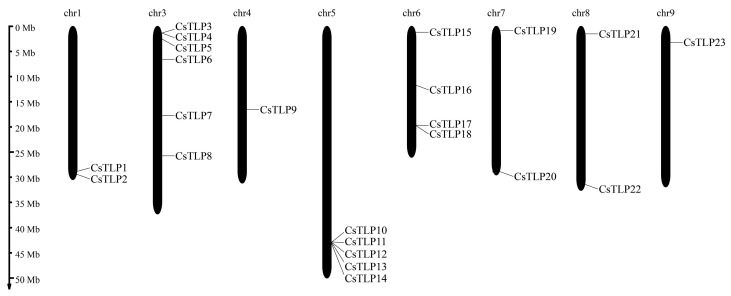
Chromosome distribution of *CsTLP* gene family members. Chromosomal locations of the 23 *CsTLPs*, which are based on the physical positions (Mb) of genes from the *Citrus sinensis* genome.

The 23 *CsTLP* genes are unevenly distributed across eight chromosomes (Figure 1 and Table S1). Chromosome 3 contains the highest number (6 genes), followed by chromosomes 5 and 6 with 5 and 4 genes, respectively. Chromosomes 1, 7, and 8 each carry 2 *CsTLP* genes, while *CsTLP9* and *CsTLP23* are located on chromosomes 4 and 9, respectively. No positive correlation was observed between chromosome length and *CsTLP* gene count.

**Figure 2:**
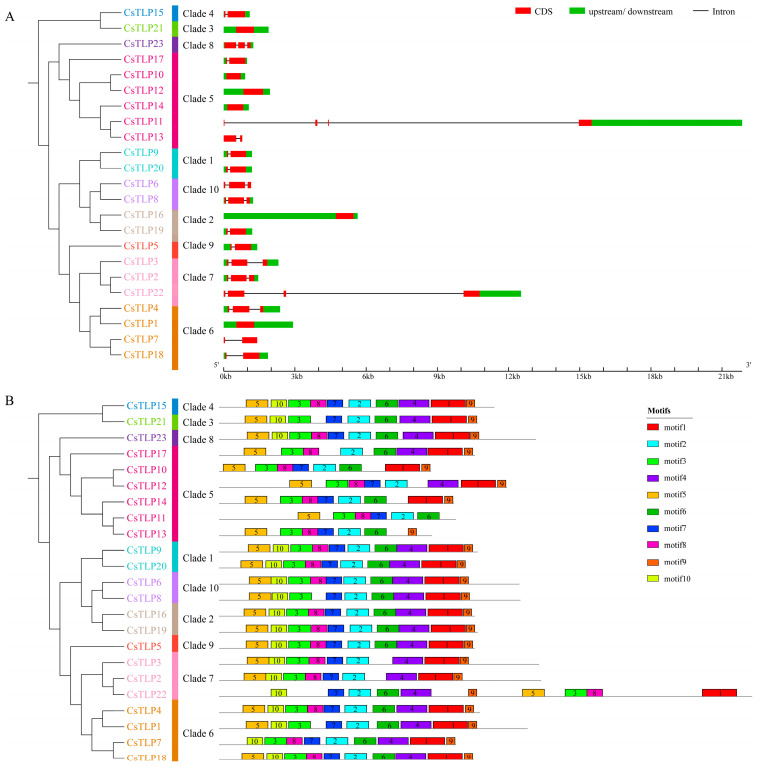
Gene structure and conserved protein motifs of *CsTLP* gene family members. (**A**) Phylogenetic relationship and exon-intron structure analysis of *CsTLP* genes. (**B**) Phylogenetic relationship and conserved motif of *CsTLP* genes.

Phylogenetic analysis using full-length protein sequences classified the 23 *CsTLP* genes into 10 clades (Figure 2). Clade 5 is the largest, containing 6 genes (*CsTLP10*/*11*/*12*/*13*/*14*/*17*), 5 of which (*CsTLP10*/*11*/*12*/*13*/*14*) reside on chromosome 5 (Figure 1). Gene structure analysis revealed intron numbers ranging from 0 to 3 per gene (Figure 2A). *CsTLP11* and *CsTLP22* contain 3 introns, 6 genes (*CsTLP2*/*3*/*4*/*6*/*8*/*23*) have 2 introns, and 9 genes (*CsTLP5*/*7*/*9*/*13*/*15*/*17*/*18*/*19*/*20*) possess a single intron. The remaining 6 are intronless. Clade 5 shows exceptional structural diversity: *CsTLP10*/*12*/*14* are intronless, *CsTLP11* has 3 introns, and *CsTLP13/17* contains one.

A total of 10 conserved motifs were identified in the *CsTLP* family (Figure 2B and Table S3). Most CsTLP proteins contain 8 or more motifs, except for CsTLP11 (6 motifs) and CsTLP13 (7 motifs), both in clade 5. Members of clade 5 lack at least 2 motifs, while proteins in other clades typically retain ≥9 motifs. Phylogenetically related members generally share conserved motif architectures in number, type, and arrangement. For instance, clade 1 members CsTLP9 and CsTLP20 exhibit identical motif patterns; clade 2 members CsTLP16 and CsTLP19 are nearly identical; clade 7 members CsTLP2 and CsTLP3 are highly similar. However, CsTLP22 in clade 7 uniquely contains motif 6 and shows distinct positioning, suggesting functional divergence among paralogs, possibly resulting from evolutionary adaptations after gene duplication.

### 2.3. Secondary and Tertiary Structure Analysis of CsTLP Proteins

Secondary structure prediction via SOPMA (Table S4) indicated that all 23 CsTLP proteins comprise α-helices, extended strands, β-sheets, and random coils. Random coils are the most abundant (53.12–64.32%), followed by extended strands (21.62–32.84%) and α-helices (5.29–16.67%) and β-sheets (2.03–7.76%), suggesting that CsTLPs are predominantly rich in random coils. Intrinsically disordered regions (IDRs) predicted using PONDR (Table S9) ranged from 7.58% to 59.87%, with an average of 28.37%. CsTLP2/3/18 exhibited IDRs > 50%, while CsTLP15 and CsTLP19 had <8%. Transmembrane helix (TMH) analysis showed that CsTLP4/5/6/11/14/18/21/23 each contain one TMH, and CsTLP2 has two, implying potential roles in antigen recognition. Notably, vacuole-localized CsTLP14, which contains a transmembrane domain, may function in pathogen defense. The remaining 14 CsTLPs lack transmembrane domains. Signal peptides were predicted in 20 CsTLPs, excluding *CsTLP10*/*11*/*12*.

Homology modeling using Phyre 2 (Figure S1, Table S4) revealed that CsTLP structures are represented by five template structures: c1z3qA_, c2ahnA_, c7p20A_, d1du5a_, and d1rqwa_. CsTLP6 and CsTLP17 are uniquely modeled with c7p20A_ and d1rqwa_, respectively. d1du5a_ covers CsTLP8 and CsTLP13; c1z3qA_ corresponds to CsTLP10/11/12/14; the remaining CsTLPs use c2ahnA_. All CsTLPs feature three domains (I, II, III) with a “V”-shaped cleft between domains I and II. Domain I consists of 7–11 anti-parallel β-sheets; domain II contains 2–6 α-helices; domain III typically includes 1–2 β-sheets and an extended loop, although some members lack β-sheets in this domain.

### 2.4. The Multiple Sequence Alignment and Phylogenetic Tree Analysis of the CsTLP Gene Family

Multiple sequence alignment of 23 CsTLP proteins (Figure S2) focused on cysteine residues, the conserved pentapeptide REDDD (R: arginine, E: glutamic acid, D: aspartic acid), and the core motif G-X-[GF]-X-C-X-T-[GA]-D-C-X(1,2)-G-X-(2,3)-C. Twenty CsTLPs contain 16 cysteine residues; CsTLP7 lacks the first cysteine; CsTLP10 and CsTLP13 have 10 and 11 cysteine, respectively. All members of the CsTLP protein family retain complete core motif, although one or two amino acid residues are substituted in CsTLP1/2/3/6/15/16/17/20/21/22, and four amino acid residues are replaced in CsTLP8. Alterations were also observed in the REDDD motif. Arginine was substituted by serine in CsTLP10 and CsTLP17; glutamic acid was replaced by asparagine in CsTLP17 and by glutamine in CsTLP21. Variations occurred in the three aspartic acid residues: the first site showed substitutions in CsTLP12/15/16/17/19/21; the second was conserved except in CsTLP10 (serine substitution); the third had substitutions in CsTLP10 (lysine) and CsTLP21 (glycine). Notably, although four amino acid residues in the core motif of CsTLP8 were substituted, the REDDD motif of CsTLP8 remains intact.

A phylogenetic tree constructed with 10 *Arabidopsis thaliana* and 23 *Citrus sinensis* TLPs (Figure S3, Table S5) grouped the 33 proteins into 10 clades. *CsTLP* distribution is uneven: clade 5 has 6 members; clades 6 and 7 contain 4 and 3, respectively; clades 1, 2, and 10 each have 2; clades 3, 4, 8, and 9 each contain one.

### 2.5. Collinearity Analysis of the CsTLP Gene Family

**Figure 3:**
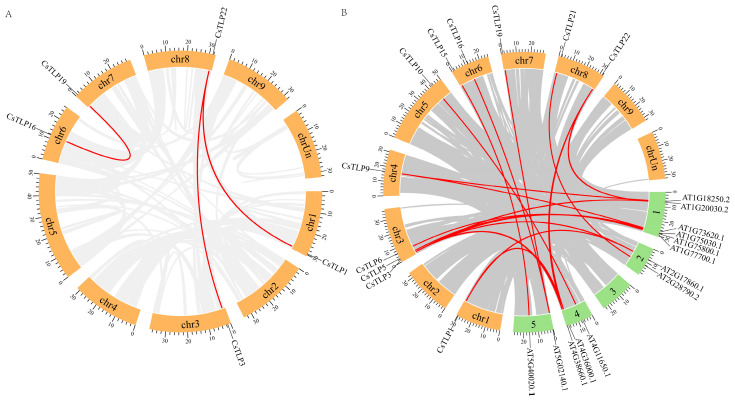
Collinearity analysis of TLP genes in the *Citrus sinensis* genome. Panel (**A**) illustrates intragenomic synteny of *CsTLP* genes. Panel (**B**) shows interspecific synteny of *TLP* genes between *Citrus Sinensis* and *Arabidopsis thaliana*.

Analysis of replication events in the *CsTLP* gene family revealed that among the 23 *CsTLPs*, three segmental duplication gene pairs were identified: *CsTLP1*/*22*, *CsTLP3*/*22*, and *CsTLP16*/*19* (Figure 3A and Table S6). In contrast, no tandem duplication events were observed in the CsTLP family. These findings suggest that such events likely played a significant role in the evolution and expansion of the *CsTLP* gene family.

To further explore the evolutionary mechanisms of the *TLP* gene family across species, duplication events were analyzed in both *Citrus sinensis* and *Arabidopsis thaliana*. The results (Figure 3B and Table S7) show 21 collinear gene pairs between the two species, involving 14 *AtTLP* genes and 11 *CsTLP* genes. This indicates that the collinearity of *TLP* genes between *Citrus sinensis* and *Arabidopsis thaliana* is considerably higher than that within *Citrus sinensis* itself.

### 2.6. Analysis of Cis-Acting Elements in the CsTLP Gene Family

**Figure 4:**
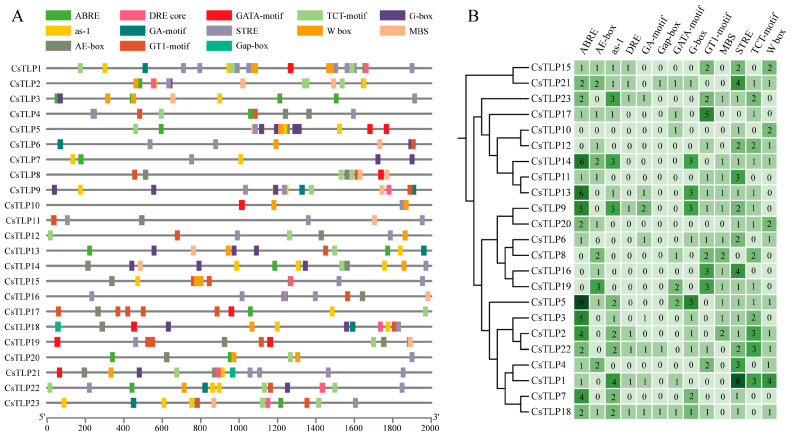
Statistical analysis of *cis*-acting elements predicted within the 2000 bp promoter regions upstream of the translation start site (ATG) of *CsTLP* genes. Panel (**A**) illustrates the distribution of 13 distinct *cis*-acting elements across the 23 *CsTLP* promoters, with each element type represented by a unique symbol; detailed information corresponding to these elements is provided in Table S8. Panel (**B**) shows the abundance of each element type in individual *CsTLP* promoters, using a color-coded grid in which both color intensity and numerical values indicate element counts.

To elucidate the roles of *cis*-acting elements in the *CsTLP* gene family, the PlantCARE database was used to predict these elements within the 2000 bp upstream sequences of the *CsTLP* transcription start sites (Figure 4 and Table S8). The analysis revealed three main categories of *cis*-acting elements: those responsive to stress, plant hormones, and light. These elements are essential for regulating gene expression regulation, as they include promoters that initiate transcription, enhancers that boost transcriptional activity, and regulatory sequences that respond to environmental signals. Notably, all *CsTLP* members contained light-responsive elements, such as AE-box, GA-Box, G-box, GATA-motif, GT1-motif, TCT-motif, and Gap-Box. Among these, GT1-motif and TCT-motif were the most abundant, with counts of 26 and 25, respectively. Stress-responsive elements included drought-inducible elements (DRE and MBS), heat shock-related elements (STRE), and wound-/pathogen-responsive W-box elements, with respective totals of 22, 43, and 19. Additionally, hormone-related elements such as abscisic acid-responsive elements (ABEB) and salicylic acid-responsive elements (as-1) were identified, numbering 55 and 28, respectively. Among the *CsTLP* genes, *CsTLP18* exhibited the highest diversity of *cis*-acting elements (11 types), while *CsTLP10* had the fewest (3 types). These results suggest that *CsTLP* gene family members may be involved in plant hormone signaling, abiotic stress responses, and biotic stress responses.

### 2.7. Analysis of Tissue-Specific Expression of the CsTLP Gene Family

We acquired transcriptome expression data for 10 tissues of *Citrus sinensis* from the CPBD database, including callus, root, leaf, calyx, early-stage ovules, late-stage ovules, peel at 120 days post-flowering, peel at 150 days post-flowering, flesh of mature fruit, and flesh of young fruit. Using the FPKM values of *CsTLPs* across these tissues, we constructed a heatmap to investigate their potential functions, as shown in Figure S4 and detailed in Table S9. The leaf exhibited the highest number of highly expressed *CsTLP* genes, with a total of 17. In callus, root, calyx, early-stage ovules, late-stage ovules, mature fruit flesh, and young fruit flesh, the number of highly expressed genes ranged from 4 to 15. Expression analysis indicated that tissue-preferential high expression among the 23 *CsTLP* genes was primarily due to a small subset of members. Notably, *CsTLP13* showed the highest expression levels in four different tissues: callus, early-stage ovules, late-stage ovules, and young fruit flesh. Similarly, *CsTLP14* displayed the highest expression in another set of four tissues: root, calyx, peel at 120 days after flowering (DAF), and peel at 150 DAF. Additionally, *CsTLP4* and *CsTLP21* exhibited the highest expression in leaf and flesh of young fruit, respectively. These results suggest that the expression of *CsTLP* genes varies widely across different tissues of *Citrus sinensis*, displaying clear tissue and organ specificity, and imply that *CsTLP4*/*13*/*14*/*21* may play important biological roles.

### 2.8. Real-Time qPCR Analysis of the CsTLP Gene Family Under Biotic Stress

**Figure 5:**
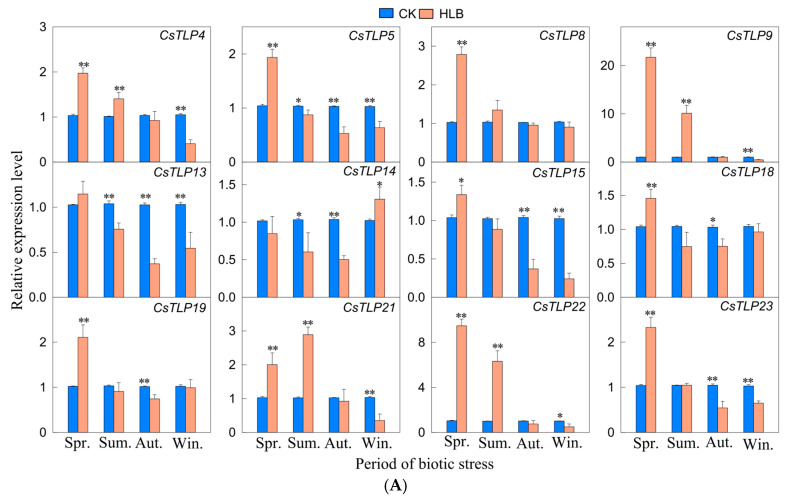

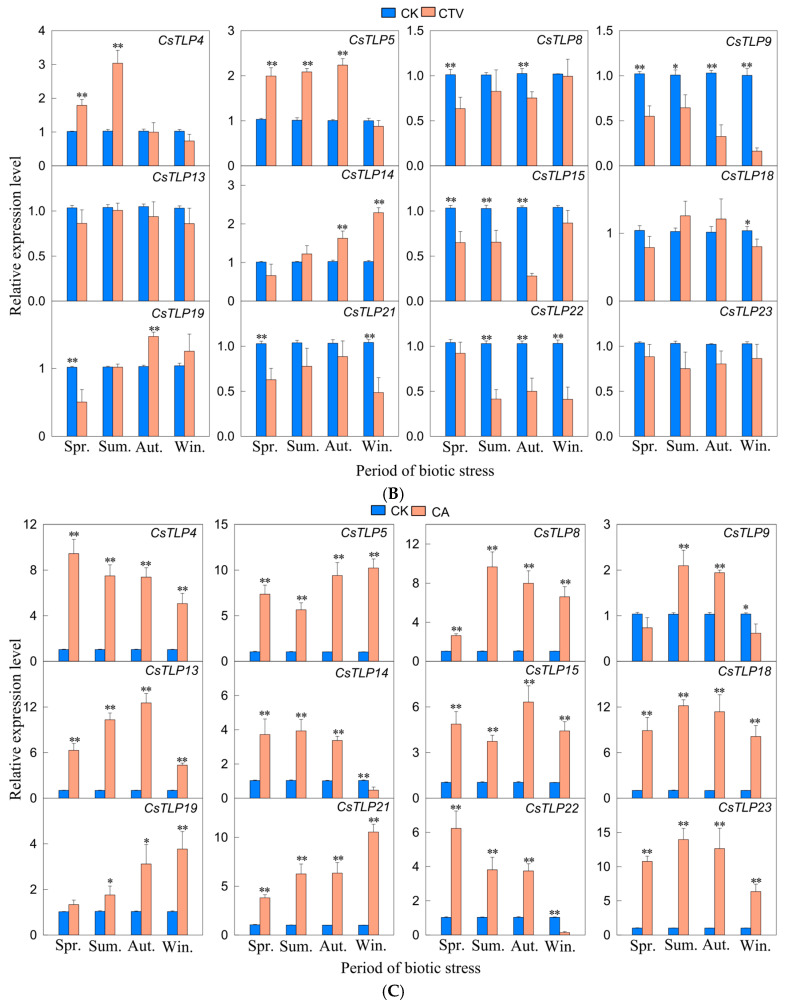
Relative expression levels of selected *CsTLP* family members in response to different biotic stresses by qPCR analysis. Plants were subjected to Huanglongbing (HLB, bacterial) (**A**), Citrus tristeza virus (CTV, viral) (**B**), and Huanglongbing (HLB, bacterial) (**C**) for spring (Spr.), summer (Sum.), autumn (Aut.), and winter (Win.). The data are plotted as means ± s.d. The error bars represent standard deviations. Significance between the control and treated conditions is carried using a two-tailed Student’s t-test. The * and ** markings represent the significance at *p*-value ≤ 0.05 and ≤ 0.01, respectively.

Drawing on the findings from phylogenetic analysis and promoter *cis*-element predictions for the *CsTLPs* family, and tissue-specific expression profiles, 12 *CsTLP* genes selected for further investigation. Using qRT-PCR, the relative expression levels of these 12 genes were assessed under HLB, CTV and CA disease stresses across four seasons (spring, summer, fall, and winter) to identify any significant differences in expression exist. Under HLB stress (Figure 5A), 10 out of 12 genes (*CsTLP4/5/8/9/15/18/19/21/22/23*) showed the highest expression in spring. Among them, *CsTLP9* and *CsTLP22* were upregulated by 21.70-fold and 9.47-fold, respectively, while the rest increased less than 3-fold. Under CTV stress (Figure 5B), *CsTLP4*/*5*/*14*/*19* showed seasonal upregulation, with *CsTLP4* increasing 3-fold in summer and the others 3-fold. Six genes (*CsTLP8*/*9*/*15*/*21*/*22*/*23*) were downregulated. Under CA stress (Figure 5C), all genes were upregulated. *CsTLP5*/*13*/*18*/*21*/*23* displayed more than 10-fold upregulation at one or two time points; eight genes (*CsTLP4*/*5*/*8*/*13*/*15*/*18*/*21*/*23*) were significantly upregulated across all seasons (2.67–13.96-fold); 3 genes (*CsTLP14*/*19*/*22*) were upregulated in three seasons; and *CsTLP9* was upregulated in summer and autumn. Five genes (*CsTLP8*/*9*/*14*/*18*/*23*) peaked in summer, while three (*CsTLP5*/*19*/*21*) peaked in winter. All genes showed significant upregulation in summer and autumn, indicating that the *CsTLP* family activates distinct response mechanisms under different biotic stresses.

## 3. Discussion

*TLPs*, as a key subfamily of PR proteins in plant defense systems, play crucial roles in the responding to both biotic stresses (e.g., pathogen infections) and abiotic stresses (e.g., drought and salinity). Their functional activity significantly enhances plant stress resistance [10,18,29]. The number of *TLP* genes varies considerably across different plant species [6]. In this study, a total of 23 *CsTLP* gene members were identified in *Citrus sinensis* through genome-wide analysis. This number is slightly fewer than in *Arabidopsis thaliana* (24 genes) [30], rice (31 genes) [17], watermelon (29 genes) [14], and grapevine (33 genes) [31], but significantly fewer than wheat (131 genes) [19], *Gossypium barbadense* (91 genes) [17], and *Phyllostachys edulis* (81 genes) [11]. These findings highlight the extensive diversity in *TLP* gene family member numbers across different plant species genomes.

With the exception of *CsTLP14*, which is localized in the vacuole, all other *CsTLP proteins* are localized in the extracellular space. This localization pattern is consistent with previous findings in plants such as cotton [32] and *Panax notoginseng* [18], where *TLPs* were extracellular. Similarly, *AmTLP25* [33] in *Ammopiptanthus mongolicus* and *AnTLP13* [10] in *Ammopiptanthus nanus* were both localized to the extracellular space through transient expression in tobacco leaves. Previous studies have suggested that extracellular TLP proteins can robustly enhance plant tolerance to various stresses, including biotic and abiotic stresses [18,29,34]. Our results support this, indicating that extracellular *TLPs* may rapidly engage in stress response mechanisms to counter external threats, playing a key role in plant defense. This provides important clues for further functional studies.

Phylogenetic analysis indicates that the *TLP* gene family members of the dicot model plant *Arabidopsis thaliana* cluster into 10 evolutionary clades [19,30,32]. Similarly, the *TLP* genes of the monocot *Oryza sativa* are distributed across 10 analogous clades [18,29,35]. This pattern is also conserved in *Citrus sinensis*, where all *TLP* genes consistently cluster into the 10 clades. Molecular evolutionary studies suggest the *TLP* family predates the divergence of monocots and dicots, originating 130–240 million years ago from 10 ancestral genes shared by both lineages [11]. Subsequently, these ancestral genes underwent asymmetric expansion through duplication events, leading to a substantial increase in *TLP* gene numbers across species. Remarkably, despite such expansion, the phylogenetic architecture of the 10 clades has remained stable, and no new clades have emerged. This stability underscores strong functional conservatism within the *TLP* gene family over long-term evolution, wherein species-specific gene proliferation occurred exclusively within the pre-existing cladal framework, without generating novel evolutionary divisions.

Gene duplication is the core mechanism behind gene family expansion, with whole-genome duplication considered a key driver of gene family expansion and a significant source of functional diversification [36,37]. Tandem duplication and segmental duplication are major contributors to gene diversity [36]. Studies on the evolution and expansion of the *TLP* gene family in plants have revealed that some species, such as ginseng, possess 6 pairs of segmentally duplicated genes but lack tandem duplications [18]. Watermelon genomes include 12 segmental duplications and one tandem duplication [14]. Thus, researchers believe that gene family expansion primarily relies on tandem duplications [18]. The grape *TLP* gene family contains 18 tandem duplications but no segmental duplications, indicating that grape *TLP* gene amplification mainly depends on tandem duplication [31]. The *TLP* gene family in melon includes 13 tandem duplications and 12 pairs of segmental duplications, suggesting that *TLP* gene amplification occurs through both pathways [38]. In this study, *CsTLP* genes exhibit no tandem duplications but have five segmental duplications, indicating that segmental duplication is the main mechanism for their expansion. Facing specific environmental pressures, species may select gene amplification strategies that are most conducive to rapid environmental adaptation [39]. Tandem duplication might be more effective and play a crucial role when there is a need to rapidly generate a large number of genes with similar functions to cope with environmental changes [40,41], as it is more efficient and can produce multiple adjacent gene copies in a short timeframe [42]. Segmental duplication may lead to the acquisition of new functions [37,43]. Thus, when facing complex and diverse environmental changes that require the coordinated action of a variety of various functional genes for adaptation, whole-genome duplication or segmental duplication may be more advantageous, offering a broader range of gene resources and functional combinations [44]. This variability may be the primary reason for the different pathways of *TLP* gene expansion among plant species. Studies have reported that citrus evolution was marked by complex and diverse environmental changes [45]. It is thus postulated that *Citrus sinensis* required the emergence of functionally distinct *CsTLP* genes acting cooperatively to adapt to these pressures, thereby favoring segmental duplication as the mechanistic outcome.

Analysis of gene structure and conserved motifs revealed that *CsTLP* members within the same branch exhibit similar gene structures, containing 0 to 3 introns, indicating a generally low intron count in *CsTLP* genes. This finding has also been confirmed in melon [38], wheat [19], and *Carya cathayensis* [9]. Genes with few or no introns can be transcribed and translated into proteins more rapidly, as they bypass the splicing process required to generate multiple protein variants, thereby shortening the response time to stress [46]. This aligns with the predominant extracellular localization of most *CsTLPs*, which facilitates rapid responses to environmental stresses, further underscoring the importance role of rapid *CsTLP* induction in defense against adverse conditions. Most CsTLP members exhibit a similar numbers and distribution pattern of conserved motifs, suggesting a degree of conservation among them. The percentage of disordered regions in CsTLP proteins ranges from 7.58% to 59.87%. These disordered regions, due to their high flexibility, can interact with diverse proteins and play key roles in biological processes such as transcription factor interactions, cell cycle regulation, and signal transduction [47]. The three-dimensional structure of CsTLP proteins in *Citrus sinensis* shows that all members consist of three domains (I, II, and III) and a “V”-shaped cleft located between domains I and II. Multiple sequence alignment results indicated that 20 CsTLP proteins contain 16 cysteine residues, forming 8 disulfide bonds. These bonds help maintain protein stability under abnormal cellular conditions, such as low pH and thermal denaturation [11]. Additionally, all CsTLP proteins contain the core conserved sequence G-X-[GF]-X-C-X-T-[GA]-D-C-X(1,2)-G-X-(2,3)-C. Furthermore 16 CsTLP proteins also possess an acidic cleavage domain composed of five highly conserved amino acids (the REDDD motif), which is thought to be associated with antifungal activity [19]. *Cis*-acting elements within promoter regions play a critical role in modulating gene expression. Bioinformatic analysis of *CsTLP* gene promoters identified several stress-responsive elements, including ABRE, as-1, MBS, and STRE, which are known to mediate responses to both biotic and abiotic stresses. Among the 23 *CsTLP* genes examined, 18 contained ABRE elements and 14 harbored as-1 elements, suggesting their potential involvement in defense-related signaling pathways. Tissue-specific expression analysis further revealed distinct spatial expression patterns of *CsTLP* genes across various tissues of *Citrus sinensis*. Fifteen genes exhibited peak expression in roots, while 17 genes were most highly expressed in leaves, indicating possible functional specialization related to organ development and environmental adaptation. These findings imply that *CsTLP* genes may not only participate in stress responses but also contribute to tissue-specific regulatory mechanisms during growth and development.

In studies investigating citrus gene responses to biotic stresses, researchers often employ extended stress treatment durations. For example, one study used RT-qPCR to analyze expression patterns of resistance-related genes (including *RLK*, *NL*, *KIN*, *TNL*, and *RLP*) at 4 and 12 months after CTV infection to monitor symptom development [48]. Similarly, researchers characterized bZIP family gene responses in *Citrus sinensis* under HLB stress by collecting leaves from plants infected with HLB for up to 4 months to examine *CsbZIP23* and *CsbZIP24* expression patterns [49]. Another study extended this temporal analysis by profiling heavy metal-associated isoprenylated plant proteins gene expression at 8, 18, 26, and 46 weeks post *Candidatus* Liberibacter asiaticus infection, revealing stage-specific expression dynamics [50]. The current study implements a comprehensive long-term stress regimen to systematically characterize *CsTLP* gene expression patterns in *Citrus sinensis* under biotic stress. Following infection with three distinct pathogens, diseased plant samples were collected at 16, 30, 43, and 55 weeks post-inoculation, corresponding to the seasons of spring, summer, autumn, and winter, respectively. This design allowed observation of *CsTLP* expression and its modulation under pathogen stress across different seasonal conditions. The multi-seasonal experimental approach facilitates an in-depth investigation of *CsTLP* gene regulatory mechanisms in plant immunity while providing new insights into their seasonal adaptation and functional specialization. Extensive studies have demonstrated that *TLPs* possess broad-spectrum antifungal activity. These proteins confer disease resistance by either enhancing β-1,3-glucanase activity or acting as xylanase inhibitors, thereby disrupting pathogen cell walls [51]. For instance, overexpression of *GhTLP1* significantly enhances *Arabidopsis thaliana* resistance to *Verticillium dahliae* [52]. In another study, researchers cloned the watermelon *ClTLP27* gene into the pET28a(+) vector and obtained the recombinant His-ClTLP27 protein through bacterial expression. Antimicrobial assays showed that this protein strongly inhibits mycelial growth of multiple fungal pathogens, including *F. oxysporum* f.sp. *niveum* race 1, *Fusarium solani* f.sp. *cucurbitae* race 1, *F. oxysporum* f.sp. *melonis*, *Fusarium verticillioides* and *Didymella bryoniae* [53]. Similarly, overexpression of the rice *Ostlp* gene in cassava (*Manihot esculenta* cv. TMS 6044) led to delayed disease progression and reduced necrotic lesion area upon *Colletotrichum gloeosporioides* infection in both leaves and stem segments, indicating enhanced fungal tolerance [54]. Additionally, transgenic wheat lines overexpressing *TaTLP1* exhibit dual resistance to common root rot caused by *Bipolaris sorokiniana* and leaf rust caused by *Puccinia triticina* [55].

In this study, under CA stress (a fungal disease), all detected *CsTLP* genes showed upregulated expression. CA is a fungal disease characterized by latent infection, with its occurrence closely linked to climatic conditions, particularly temperature and humidity and tree vigor. Generally, high temperatures and abundant rainfall during spring and summer create peak periods for infection and disease development, although young trees and weakened plants may also experience severe symptoms in autumn and winter [25]. In this experiment, all 12 *CsTLPs* analyzed showed significant upregulation in both summer and autumn, with 11 *CsTLPs* exhibiting a more than threefold increase in relative expression compared to the control group. Since the samples were collected from young trees, 9 *CsTLPs* were significantly upregulated even in winter, indicating that multiple genes respond strongly to CA infection across all seasons. *CsTLPs* are well established to possess specific antifungal activity, leading to the hypothesis that citrus *TLPs* are highly expressed in response to anthracnose infection, thereby contributing to disease resistance. Furthermore, expression patterns varied among the genes: some were consistently upregulated throughout the year, while others showed seasonal specificity, being highly expressed only in certain seasons. In the resistant tomato cultivar ‘S-55’, *SlTLP5* and *SlTLP6* were up-regulated under both fungal and bacterial pathogens, and their overexpression enhanced resistance to all five pathogens tested, with stronger effects against fungi, suggesting a role in bacterial defense [20]. Under bacterial HLB stress, ten *CsTLPs* showed highest expression in spring; *CsTLP9* and *CsTLP22* were upregulated by 21.70-fold and 9.47-fold, respectively, while others increased less than threefold. The causal agent of Citrus HLB is *Candidatus* Liberibacter asiaticus, an obligate parasitic bacterium colonizing the phloem of citrus plants. During spring, as temperatures rise, the leaf yellowing symptoms of HLB tend to alleviate, and the vigor of infected trees increases, promoting vigorous sprouting of new shoots [56]. Concurrently, the elevated expression of *CsTLPs* in leaves is hypothesized to be associated with the mitigation of leaf yellowing in diseased plants. This study also explored changes in the relative expression of *CsTLPs* under viral disease CTV stress. Although previous research has suggested that *TLP* genes may confer resistance to plant viral diseases, our findings revealed that only *CsTLP4* exhibited a threefold increase in expression during summer, while the upregulation of other genes remained below this threshold. Moreover, six genes showed decreased expression. These results indicate that the response of *CsTLPs* to the viral pathogen CTV is relatively limited.

## 4. Materials and Methods

### 4.1. Plant Materials and Treatment

On 25 November 2023, one-year-old Shatian pomelo (*Citrus maxima*) seedlings were used as rootstocks. Scions were collected from previously tested *Citrus sinensis* mother plants that had been previously tested and confirmed to be infected with citrus HLB, CA and CTV. These mother plants are preserved and cultivated at the National Navel Orange Engineering Research Center. The selected scions exhibited uniform vigor and plump buds. Non-pathogenic scions were grafted as a control (CK). All experimental plants were cultivated in the greenhouse of the National Citrus Engineering Research Center at Gannan Normal University. Pathogenic testing was performed on the germinated spring shoots in early March 2024. The detection methods were as follows: HLB was detected via qRT-PCR [57], CA was confirmed by PCR amplification using universal fungal primers ITS1 and ITS4 [58], and CTV was identified by RT-PCR targeting the coat protein (CP) gene of CTV [59]. On 15 March, 17 June, 16 September, and 13 December 2024 corresponding to spring, summer, autumn, and winter, respectively, leaves were collected from infected plants, with three biological replicates per sample. Leaves from healthy (disease-free) plants were also collected as controls. All samples were rapidly frozen in liquid nitrogen and stored at −80 °C in an ultra-low temperature freezer for subsequent analysis.

### 4.2. Identification of the CsTLP Gene Family

The whole-genome sequence of Citrus sinensis was obtained from the CPBD database (http://citrus.hzau.edu.cn/index.php, accessed on 1 October 2023) [60]. Using the protein domain PF00314 as a query, we applied HMMER 3.0 to identify the TLP gene family in the Citrus sinensis genome database Subsequent protein domain analysis was performed using CDD (https://www.ncbi.nlm.nih.gov/cdd/?term=, accessed on 1 October 2023) and SMART (http://smart.embl-heidelberg.de/, accessed on 1 October 2023). Only sequences containing the TLP domain (PF00314) were retained as final CsTLP candidates. The physicochemical properties of CsTLP proteins, including amino acid length, molecular weight (MW), and isoelectric point (pI) were analyzed using ExPASy ProtParam (http://web.expasy.org/protparam, accessed on 1 October 2023). Additionally, subcellular localization predictions were conducted using Cell-PLoc 2.0 (http://www.csbio.sjtu.edu.cn/bioinf/Cell-PLoc-2/, accessed on 1 October 2023).

### 4.3. Chromosomal Localization, Gene Structure, and Conserved Motifs of the CsTLP Gene Family

The chromosomal positions of the *CsTLP* genes were mapped using M2G2 (http://mg2c.iask.in/mg2c_v2.0, accessed on 1 October 2023), based on the chromosome lengths of *Citrus sinensis* and the location information of each *CsTLP* gene. Subsequently, TBtools was used to extract the exon and intron positions of *CsTLP* members. These data were submitted to GSDS (http://gsds.gao-lab.org/, accessed on 1 October 2023) to generate the gene structure diagram of *CsTLP* genes. Furthermore, conserved motifs in the *CsTLP* protein sequences were identified using MEME (http://meme-suite.org/tools/meme, accessed on 1 October 2023) with the following parameter settings: the number of motifs was set to 10, and the motif amino acid length ranged from 6 to 50.

### 4.4. Prediction of Secondary and Tertiary Structures of CsTLP Proteins

The secondary structure of *CsTLP proteins* was predicted using the SOPMA website (https://npsa-prabi.ibcp.fr/cgi-bin/npsa_automat.pl?page=npsa_sopma.html, accessed on 1 October 2023). The tertiary structure was predicted with the PHYRE2 website (http://www.sbg.bio.ic.ac.uk/phyre2/html/page.cgi?id=index, accessed on 1 October 2023) and visualized using PyMOL 2.5.5. Transmembrane domains of the CsTLP proteins were predicted using DeepTMHMM (https://services.healthtech.dtu.dk/services/DeepTMHMM-1.0/, accessed on 1 October 2023). Signal peptides were predicted with SignalP 6.0 (https://services.healthtech.dtu.dk/services/SignalP-6.0/, accessed on 1 October 2023). Disordered regions were predicted using PONDR (http://www.pondr.com/, accessed on 1 October 2023).

### 4.5. Multiple Sequence Alignment, Phylogenetic Tree, and Collinearity Analysis of the CsTLP Gene Family

All CsTLP protein sequences were aligned using MEGA 7.0 and visualized with Jalview. To examine the evolutionary relationships between CsTLP proteins and their *Arabidopsis thaliana* homologs, ten TLP proteins from various branches of the A. thaliana TLP family were selected. A phylogenetic tree was then constructed with MEGA 7.0 using the neighbor-joining method with 1000 bootstrap replicates. Additionally, to investigate potential gene duplication events in the evolution of the *CsTLP* gene family and assess their homology with the *Arabidopsis thaliana* genome, collinearity analysis was conducted using TBtools. This analysis involved comparisons within *Citrus sinensis*, as well as between *Citrus sinensis* and *Arabidopsis thaliana*.

### 4.6. Cis-Acting Elements and Tissue Specificity Analysis of the CsTLP Gene Family

The 2000 bp sequences upstream of the transcription start sites of *CsTLP* family members were submitted to the PlantCARE database (http://bioinformatics.psb.ugent.be/webtools/plantcare/html/, accessed on 1 October 2023) for *cis*-acting regulatory element analysis. The results were visualized using TBtools v2.1. Transcript abundance data (FPKM values) of *CsTLP* genes from seven tissues of *Citrus sinensis* including callus tissue, roots, ovules, leaves, fruits, peels, and sepals were obtained from the CPBD. Based on these data, heat maps were generated and visualized using TBtools.

### 4.7. RNA Extraction and qRT-PCR Analysis

Based on the phylogenetic tree of the *CsTLP* gene family and their tissue-specific expression patterns, 12 *CsTLP* genes were selected for further analysis. The qRT-PCR primer sequences were designed using Premier 5 software (Table S10). Total RNA was extracted from both healthy and diseased *Citrus sinensis* leaves using the Trizol method. Subsequently, reverse transcription was performed with the cDNA first-strand synthesis kit (SIMGEN, Hangzhou, China). The citrus ACTB gene was used as the internal reference. qRT-PCR reactions were prepared using the 2×SYBR Green PCR Mix kit (SIMGEN, Hangzhou, China) and conducted on a LightCycler 480 instrument (Roche, Basel, Switzerland). Each experimental group included three biological replicates, with each biological replicate consisting of three technical replicates. The relative expression levels of the target genes were calculated using the comparative Ct method [61]. Statistical significance of differences was evaluated by Duncan’s new multiple range test, and the resulting data were visualized with Sigmaplot 14.0.

## 5. Conclusions

This study identified 23 *TLP* genes in *Citrus sinensis*. Most CsTLP proteins were found to be unstable and predominantly localized in the extracellular space. The genes were unevenly distributed across chromosomes and classified into 10 clades, with clade 5 exhibiting structural variations that suggest functional divergence. Protein structure predictions revealed a dominance of random coils, and many members were identified to contain signal peptides or transmembrane domains, indicating their potential involvement in pathogen defense mechanisms. Under biotic stress, *CsTLPs* exhibited pathogen-specific responses, with significant upregulation exceeding 10-fold in cases of CA infection (e.g., *CsTLP5*/*13*/*18*/*21*/*23*), highlighting their potential role in disease resistance. These findings demonstrate that *CsTLPs* can effectively respond to CA infection throughout all four seasons. Since *TLP* genes have been shown to possess antifungal activity, it is reasonable to infer that the high expression of *CsTLPs* in response to CA infection contributes to plant resistance. Previous studies have reported that citrus can utilize endogenous compounds to enhance its antifungal capacity [62]. Therefore, TLP proteins are promising candidates for developing novel broad-spectrum antimicrobial agents, offering a potential strategy for controlling fungal plant diseases. This study also provides an important theoretical basis for improving molecular antibacterial breeding in *Citrus sinensis*.

## Figures and Tables

**Table 1:** Nomenclature, Gene ID, peptide lengths, molecular weights (MW), theoretical isoelectric points (PI), instability index (II) aliphatic indices (AI), Grand Average of Hydropathicity (GRAVY) and subcellular localization of *CsTLP* family members.

Gene Name	Gene ID	AA	MW (kDa)	pI	II	AI	GRAVY	Subcellular Localization
*CsTLP1*	Cs_ont_1g028250.1	296	30.67	4.66	44.14	71.01	0.145	Extracellular
*CsTLP2*	Cs_ont_1g028270.1	309	32.22	4.50	51.85	59.77	−0.072	Extracellular
*CsTLP3*	Cs_ont_3g002150.1	307	32.62	4.69	47.53	61.99	−0.066	Extracellular
*CsTLP4*	Cs_ont_3g002160.1	250	26.20	4.93	41.26	59.76	−0.089	Extracellular
*CsTLP5*	Cs_ont_3g004110.1	245	25.11	4.50	33.53	67.71	0.178	Extracellular
*CsTLP6*	Cs_ont_3g009770.1	288	30.83	8.59	37.51	73.09	0.085	Extracellular
*CsTLP7*	Cs_ont_3g021910.1	227	23.34	4.60	42.39	65.86	0.004	Extracellular
*CsTLP8*	Cs_ont_3g026270.1	289	31.00	7.37	44.15	70.90	−0.121	Extracellular
*CsTLP9*	Cs_ont_4g012400.1	248	26.75	9.38	54.46	76.41	0.036	Extracellular
*CsTLP10*	Cs_ont_5g040270.1	203	21.88	5.73	28.24	64.43	−0.186	Extracellular
*CsTLP11*	Cs_ont_5g040300.1	227	24.88	6.58	34.60	61.06	−0.365	Extracellular
*CsTLP12*	Cs_ont_5g040310.1	276	30.02	5.92	28.84	60.07	−0.167	Extracellular
*CsTLP13*	Cs_ont_5g040320.1	204	21.98	8.24	34.43	78.48	−0.072	Extracellular
*CsTLP14*	Cs_ont_5g040330.1	225	24.54	8.13	24.13	63.29	−0.193	Vacuole
*CsTLP15*	Cs_ont_6g002020.1	264	29.07	6.09	57.99	71.70	−0.025	Extracellular
*CsTLP16*	Cs_ont_6g017000.1	245	25.70	7.82	40.96	75.22	0.079	Extracellular
*CsTLP17*	Cs_ont_6g022360.1	245	26.51	4.92	46.79	72.04	−0.213	Extracellular
*CsTLP18*	Cs_ont_6g022370.1	244	25.69	5.81	34.85	55.74	−0.145	Extracellular
*CsTLP19*	Cs_ont_7g000750.1	248	26.53	8.16	38.46	71.61	0.094	Extracellular
*CsTLP20*	Cs_ont_7g028380.1	236	25.11	4.94	41.34	65.34	−0.143	Extracellular
*CsTLP21*	Cs_ont_8g002970.1	248	26.73	8.16	55.84	70.04	−0.037	Extracellular
*CsTLP22*	Cs_ont_8g027660.1	512	53.75	6.44	44.91	60.66	−0.107	Extracellular
*CsTLP23*	Cs_ont_9g005100.1	304	32.36	5.05	41.12	68.36	0.036	Extracellular

## Data Availability

Data is contained within the article and Supplementary Materials.

## Supplementary Materials

The following supporting information can be downloaded at: https://www.mdpi.com/article/10.3390/ijms262010133/s1.

## Abbreviations

The following abbreviations are used in this manuscript:

TLPs	Thaumatin-like proteins
PR	Pathogenesis-related
CDS	Coding sequence
GA	Gibberellic acid
aa	Amino acid
MW	Molecular weight
pI	Isoelectric point
NLS	N-terminal nuclear localization signal
ORF	Open reading frame
qRT-PCR	Quantitative real-time polymerase chain reaction
ZF	Zinc finger
SAR	Systemic acquired resistance
HR	Hypersensitive response
HLB	Citrus huanglongbing
CTV	Citrus tristeza virus
CA	Citrus Anthracnose
IDRs	Intrinsically disordered regions
TMH	Transmembrane helix
AREB	Abscisic acid response elements
HIPPs	Heavy metal-associated isoprenylated plant proteins
UTR	Untranslated region
REDDD	Arginine, glutamic acid, and three aspartic acid residues
