# Supplementary figures and images for: Genome-Wide Identification and Biotic Stress Responses of TLP Gene Family in Citrus sinensis
Source: Int J Mol Sci. 2025 Oct 18;26(20):10133. doi: 10.3390/ijms262010133 (PMC12564699; doi:10.3390/ijms262010133)

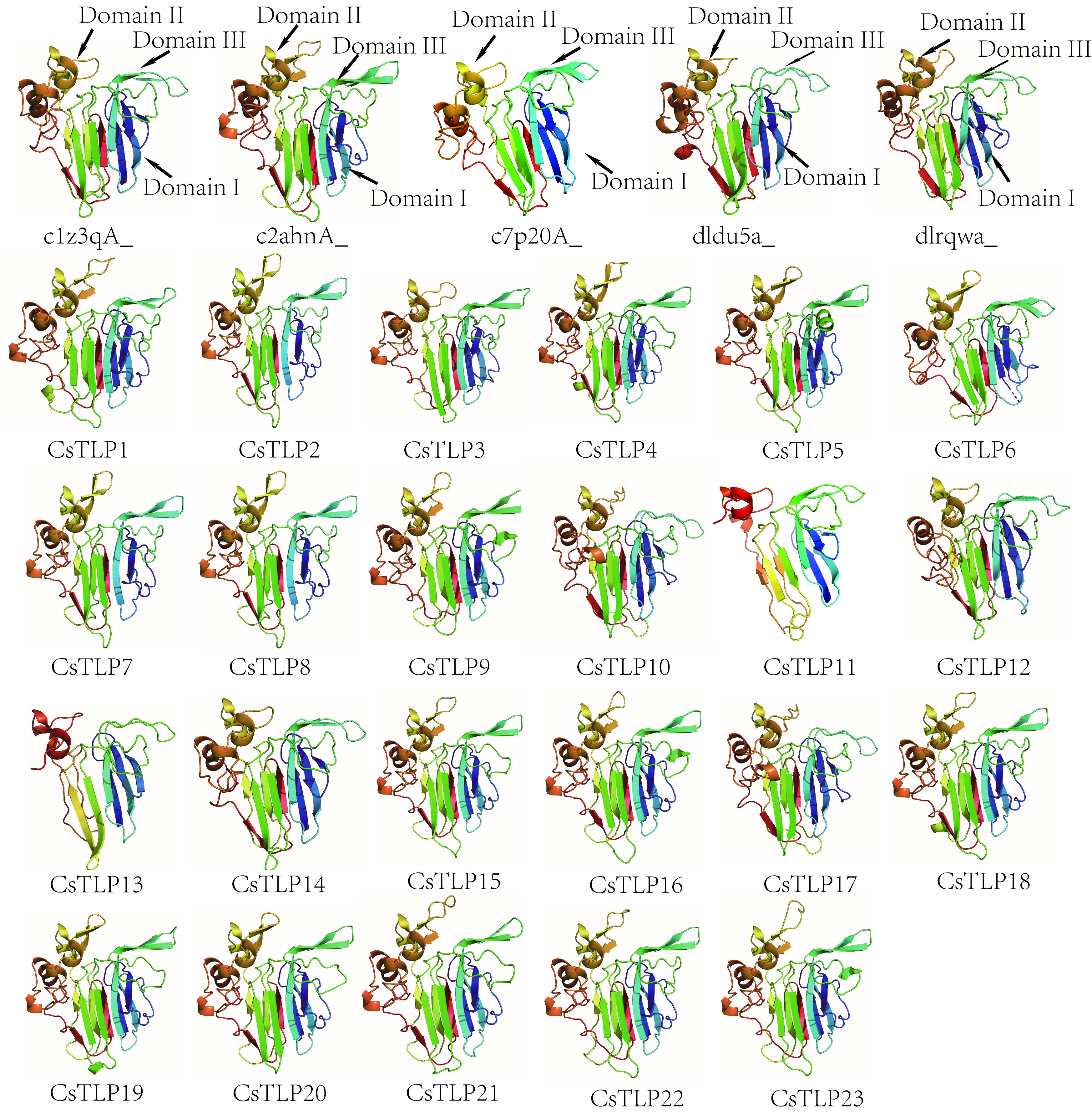

Supplement: Supplementary file 1 [file ijms-26-10133-s001.zip › Figure S1 Homology modeling of the CsTLP tertiary structure.tif]

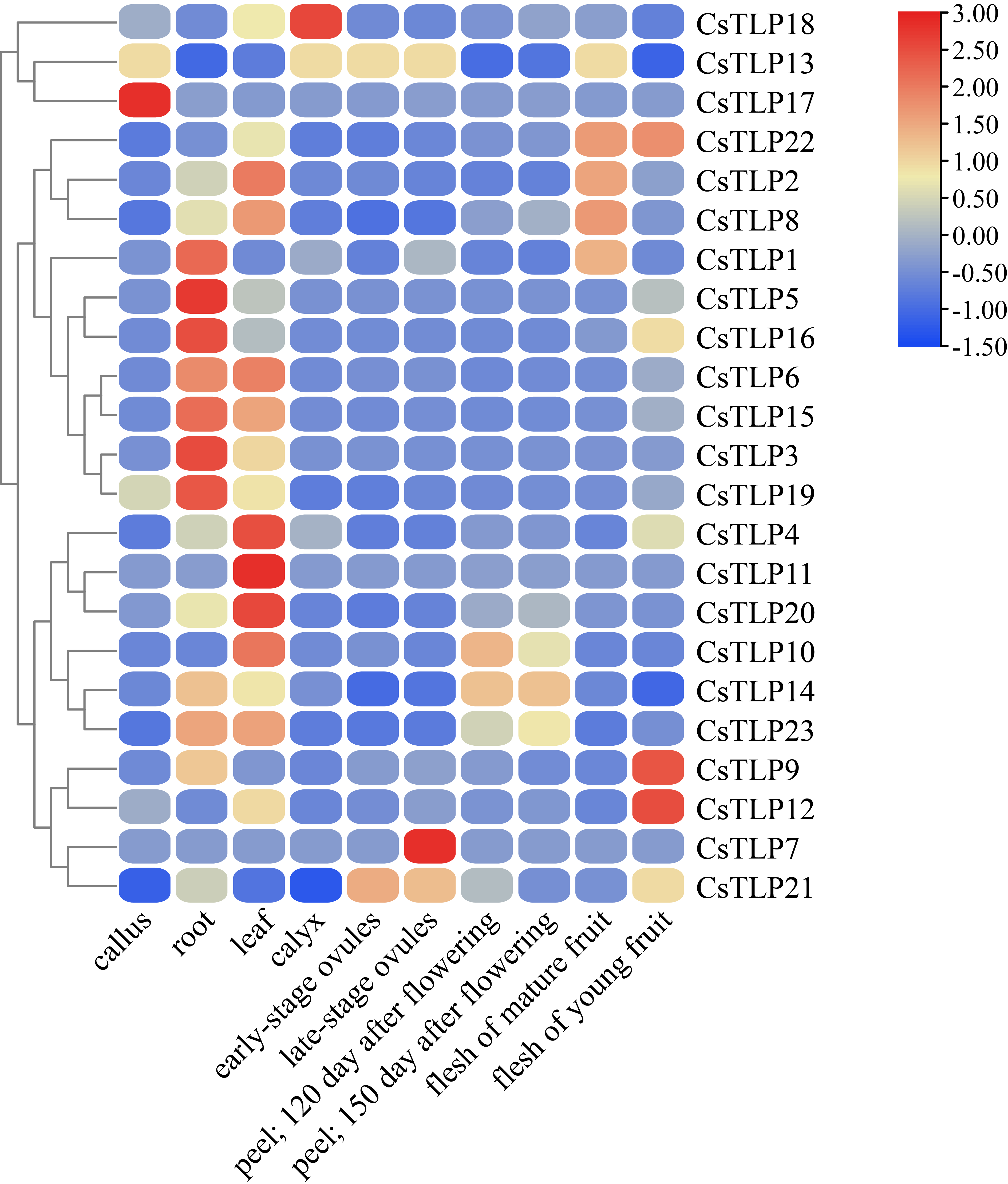

Supplement: Supplementary file 1 [file ijms-26-10133-s001.zip › Figure S4 Expression patterns of CsTLP gene family members in various Citrus sinensis tissues.tif]
